# Current News

**Published:** 1902-11

**Authors:** 


### Current News.

### BOARD OF DENTAL EXAMINERS OF PENNSYLVANIA.

THE Board of Dental Examiners of Pennsylvania will conduct examinations simultaneously in Philadelphia and Pittsburg, December 16 to 19, 1902. For application papers or further information, address Hon. James W. Latta, secretary Dental Council, Harrisburg, Pa.

G. W. KLUMP,
*Secretary.*

---

### COLORADO STATE BOARD OF DENTAL EXAMINERS.

THE Board of Dental Examiners of the State of Colorado will meet in Denver, Col., Tuesday, December 2, 1902, at nine A.M., for examination of applicants for license to practise dentistry in Colorado.

In addition to written and oral examination, applicants must supply their own patients, instruments, and materials, and come prepared to do practical work under the supervision of the board, which will pass upon suitable selection of cavities.

All applications must be completed prior to December 2.

For application blanks and information, address

H. F. HOFFMAN,
*Secretary.*

611 CALIFORNIA BUILDING, DENVER, COL.

